# Veteran, Primary Care Provider, and Specialist Satisfaction With Electronic Consultation

**DOI:** 10.2196/medinform.3725

**Published:** 2015-01-14

**Authors:** Keri L Rodriguez, Kelly H Burkitt, Nichole K Bayliss, Jennifer E Skoko, Galen E Switzer, Susan L Zickmund, Michael J Fine, David S Macpherson

**Affiliations:** ^1^VA Pittsburgh Healthcare SystemCenter for Health Equity Research and PromotionPittsburgh, PAUnited States; ^2^University of Pittsburgh School of MedicineDivision of General Internal Medicine, Department of MedicinePittsburgh, PAUnited States; ^3^VA Pittsburgh Healthcare SystemVeterans Engineering Resource CenterPittsburgh, PAUnited States; ^4^Chatham UniversityDepartments of Psychology and Social Work/CriminologyPittsburgh, PAUnited States; ^5^VA Pittsburgh Healthcare SystemFacility Management ServicePittsburgh, PAUnited States; ^6^University of PittsburghDepartment of PsychiatryPittsburgh, PAUnited States; ^7^University of PittsburghClinical and Translational Science InstitutePittsburgh, PAUnited States; ^8^Veterans Integrated Service Network 4 (VISN 4) Healthcare NetworkPittsburgh, PAUnited States

**Keywords:** access, rural health, referral and consultation, patient satisfaction, veterans

## Abstract

**Background:**

Access to specialty care is challenging for veterans in rural locations. To address this challenge, in December 2009, the Veterans Affairs (VA) Pittsburgh Healthcare System (VAPHS) implemented an electronic consultation (e-consult) program to provide primary care providers (PCPs) and patients with enhanced specialty care access.

**Objective:**

The aim of this quality improvement (QI) project evaluation was to: (1) assess satisfaction with the e-consult process, and (2) identify perceived facilitators and barriers to using the e-consult program.

**Methods:**

We conducted semistructured telephone interviews with veteran patients (N=15), Community Based Outpatient Clinic (CBOC) PCPs (N=15), and VA Pittsburgh specialty physicians (N=4) who used the e-consult program between December 2009 to August 2010. Participants answered questions regarding satisfaction in eight domains and identified factors contributing to their responses.

**Results:**

Most participants were white (patients=87%; PCPs=80%; specialists=75%) and male (patients=93%; PCPs=67%; specialists=75%). On average, patients had one e-consult (SD 0), PCPs initiated 6 e-consults (SD 6), and VAPHS specialists performed 17 e-consults (SD 11). Patients, PCPs, and specialty physicians were satisfied with e-consults median (range) of 5.0 (4-5) on 1-5 Likert-scale, 4.0 (3-5), and 3.5 (3-5) respectively. The most common reason why patients and specialists reported increased overall satisfaction with e-consults was improved communication, whereas improved timeliness of care was the most common reason for PCPs. Communication was the most reported perceived barrier and facilitator to e-consult use.

**Conclusions:**

Veterans and VA health care providers were satisfied with the e-consult process. Our findings suggest that while the reasons for satisfaction with e-consult differ somewhat for patients and physicians, e-consult may be a useful tool to improve VA health care system access for rural patients.

## Introduction

The Department of Veterans Affairs (VA) operates the largest integrated health care delivery system in the United States [1]. Many of the veterans served by the VA live in rural areas [2]. For example, within the VA system approximately 36% of the total enrolled veteran population and 15% of those seen for at least one service-connected disability are from rural or highly rural areas [2]. Rural areas present challenges to providing care to veterans from specialists that are almost exclusively based in a smaller number of large medical centers in urban areas [1].

One method to improve access to specialty care for rural veterans is through electronic consultations or e-consults, a telehealth modality. The VA’s Office of Specialty Care Services/Office of Specialty Care Transformation launched an e-consult initiative to improve access to and delivery of specialty care that are veteran-centered, efficient, and evidence- and team-based [3]. In December 2009, the VA Pittsburgh Healthcare System (VAPHS) implemented an e-consult program designed to provide primary care providers (PCPs) access to specialists to enhance communication about short-term diagnostic and therapeutic management issues [3]. These e-consults, requested by the veteran’s PCPs and completed by specialists at the affiliated VA medical center, provide an opportunity for PCPs who manage patients at remote Community Based Outpatient Clinics (CBOCs) or medical centers to obtain a consultation from a specialty care provider without requiring their patients to have a face-to-face encounter with the specialist. 

PCPs in the western Pennsylvania region (associated with VA Pittsburgh) were sent a letter by the chief medical officer of the Veterans Integrated Service Network (VISN) 4 introducing a new program called “E consults” with a subset of medical (ie, cardiology, diabetes/endocrinology, renal, women’s health) and surgical (ie, neurosurgery, orthopedics) specialty areas. They were informed that e-consults are specialty consultations you can receive without the patient needing to travel for a face-to-face visit with the specialist and that they offer your patients and you more convenient access to selected specialists. They were also notified that e-consults are best suited for questions about short-term diagnostic and therapeutic issues, but that they can also be used for specialist advice on what tests are needed in advance of a face-to-face specialist visit or for ongoing advice on how best to manage a chronic condition, such as a chronic kidney disease. PCPs were also provided the following e-consult information: (1) a script to use when speaking to veterans; (2) an informational brochure to give veterans; and (3) operational guidelines for e-consults developed by a group of PCPs and specialists. The operational guidelines instructed PCPs to select the “E consult” option from the Computerized Patient Record System (CPRS) consult tab and complete the template for veterans for whom the e-consult option appeared to be appropriate. For example, screenshots of the CPRS template for a cardiology e-consult are presented in Appendix A. The appropriate specialist at the VA medical center would then review the patient’s VA electronic medical records in the Computerized Patient Record System (CPRS), a fully integrated electronic health record that allows a VA provider nationwide, including a referring provider and specialist, to access and view a veteran’s entire medical record; all clinical information including progress notes, laboratory tests, radiology results, discharge summaries, other consultant reports, pathology results, and surgery reports are available [4]. The specialist would then “complete” the consult by providing an assessment via a progress note entered in the medical record, requesting additional diagnostic testing, scheduling a face-to-face visit with the veteran at the medical center, or other appropriate follow-up care. Veterans who alternatively requested face-to-face specialist care had their request honored.

The goals of this quality improvement project evaluation were to: (1) assess satisfaction with this e-consult program, and (2) identify perceived facilitators of and barriers to e-consult utilization.

## Methods

### Setting and Participants

Between August-October 2010, we conducted individual semistructured telephone interviews with veteran patients, CBOC or rural medical center PCPs, and VAPHS medical center specialty physicians who had used the e-consult process. To minimize recall bias, only veterans who participated in an e-consult during June-August 2010 were eligible to participate. We also interviewed providers who participated in one or more e-consults between December 2009 to August 2010. We attempted to contact all eligible patients (n=30) and PCPs (n=22); we stopped data collection when we reached our target sample size (n=15 patients and PCPs). Due to the limited number of eligible specialty physicians, we attempted to interview all 6 eligible specialty physicians and were unable to reach 2 during our data collection period. Our sample size was guided by the qualitative principle of “saturation,” a process by which researchers collect and analyze data until no new themes are generated. It has been suggested that saturation can be reached with 12-15 participants within each group [5].

This quality improvement evaluation project was approved with a waiver of informed consent by the Institutional Review Board at the VAPHS.

### Interview Content

Initially, all participants were asked to describe their e-consult experience in their own words (see Appendix B). Then, using additional open-ended questions, participants were asked to describe perceived barriers and facilitators to participating in the e-consult process. Next, respondents were asked to rate their satisfaction with e-consultation across eight domains: overall satisfaction, quality, time, access, safety, expectations, confidence, and intention to use e-consult in the future, using a 5-point Likert-type scale ranging from “completely agree” to “completely disagree”. Participants first rated their satisfaction in each domain and then answered the open-ended question: “What are the important things that made you respond in this way about your satisfaction with the e-consult process?” Finally, participants were asked, “Given all of the things we have just talked about, which one thing is the most important to your satisfaction with the e-consult process?”

### Data Coding and Analysis

We created descriptive summaries of the characteristics of project participants and their use of e-consults, as well as the quantitative Likert-type items regarding satisfaction with the e-consult process for each of the eight specific domains. We then identified key themes from the qualitative data for each of the eight individual satisfaction domains, as well as perceived barriers and facilitators to participating in the e-consult process.

Qualitative analysis began with codebook construction using a modified grounded theory methodology to provide rich information about veteran, PCP, and specialty physician satisfaction with the e-consult process [6,7]. The primary coder started the process by reading the transcripts from interviews with patients, PCPs, and specialists for emerging themes. The codes were recorded in a master file, which then became the basis for the final analysis. The resulting codebook was finalized and applied to all interviews by the primary coder and a co-coder per established standards in qualitative analysis [8]. During this coding process, the coders also tabulated any new or emerging themes that appeared during the course of reviewing the transcripts. This process of coding enabled the project team to maintain narrative coherence in the qualitative coding.

When there were no further changes to the coding scheme, we tested its reliability by coding all of the transcripts independently, comparing the results, and calculating an inter-rater reliability coefficient [9]. For each code, the kappa values, obtained from a statistical method of inter-rater agreement [9], ranged in value from .79 to .95. The kappa values for each code were as follows: overall satisfaction (.95), quality (.90), time (.79), access (.85), safety (.83), expectations (.83), confidence (.80), intent to use e-consult in the future (.92), most important domain (.91), barriers (.86), and facilitators (.86). We achieved a kappa value of .80 or greater, or “almost perfect” [9] on all but one code (time=.79).

## Results

### Participant Characteristics

Semistructured interviews were completed by 15 patients, 15 CBOC or rural medical center PCPs, and 4 VAPHS physician specialists. Veteran patients were primarily white (87%), male (93%), and had a mean age of 63 (SD 12). On average, veteran patients rated their health as fair (2+1 on 1-5 Likert-scale), and reported receiving care from the VA for a mean year of 10 (SD 10) (Table 1).

PCPs were primarily white (80%), male (67%) physicians (73%), with a mean age of 46(SD 10) who were practicing medicine for a mean year of 15 (SD 8), and 7 (SD 5) years practicing within the VA (Table 2).

Specialists were primarily white (75%), male (75%) physicians (100%), with a mean age of 55 (SD 13), and an average of 25 (SD 14) years practicing medicine and 18 (SD 16) years practicing within the VA. The four specialists interviewed were in the fields of cardiology, diabetes/endocrinology, nephrology/renal care, and orthopedics (Table 2).

Patients (Table 1) and PCPs (Table 2) were from a wide range of CBOCs or rural medical centers in the western region of Pennsylvania, with patients coming from 9 unique facilities and PCPs from 14 unique facilities.

**Table 1:** Patient characteristics.

Patient characteristics		Patient (N=15) n (%)
**Age**		
	Mean (SD)	63 (12)
**Gender**		
	Male	14 (93)
	Female	1 (7)
**Race**		
	White	13 (87)
	Black/African American	2 (13)
**Years receiving care at VA**		
	Mean (SD)	10 (10)
**CBOC/Medical Center (western region)**		
	Cranberry Township	1 (7)
	Crawford (Meadville)	4 (27)
	DuBois (Clearfield County)	1 (7)
	Erie	2 (13)
	Mercer County (Hermitage)	1 (7)
	Monongalia County	2 (13)
	Tucker County (Parsons)	2 (13)
	Venango County	1 (7)
	Wood County (Parkersburg)	1 (7)
**Self-rated general health**		
	Excellent (5)	0 (0)
	Very good (4)	1 (7)
	Good (3)	6 (40)
	Fair (2)	3 (20)
	Poor (1)	5 (33)
**Self-rated health compared to 1 year ago**		
	Much better now (5)	2 (13)
	Somewhat better now (4)	3 (20)
	About the same (3)	8 (53)
	Somewhat worse now (2)	0 (0)
	Much worse now (1)	2 (13)
**Marital status**		
	Never married	1 (7)
	Married or living as married	12 (80)
	Widowed	2 (13)
**Education**		
	Less than 9^th^ grade	1 (7)
	9^th^-12^th^ grade, no diploma	0 (0)
	High school graduate/GED	10 (67)
	Trade/vocational school	1 (7)
	Some college, no degree	0 (0)
	Associate’s degree	0 (0)
	Bachelor’s degree	3 (20)
**Employment status**		
	Employed part-time	3 (20)
	Not currently employed	2 (13)
	Retired	10 (67)
**Residence**		
	Own	12 (80)
	Rent	1 (7)
	Live with others, rent free	2 (13)
**Household income**		
	Less than $10,000 per year	1 (7)
	$10,000-$19,999 per year	6 (40)
	$20,000-$34,999 per year	2 (13)
	$35,000-$49,999 per year	1 (7)
	$50,000 or greater per year	3 (20)
	Refused	2 (13)

**Table 2:** Primary Care Provider (PCP) and Specialty Physician Characteristics.^a^

Characteristics		PCP (N=15) n (%)	Specialist (N=4) n (%)
**Age**			
	Mean (SD)	46 (10)	55 (13)
**Gender**			
	Male	10 (67)	3 (75)
	Female	5 (33)	1 (25)
**Race**			
	White	12 (80)	3 (75)
	Black/African American	0 (0)	0 (0)
	Asian	3 (20)	1 (25)
**Years in practice (overall)**			
	Mean (SD)	15 (8)	25 (14)
**Years in practice at VA**			
	Mean (SD)	7 (5.0)	18 (16)
**Primary care provider type**			
	Physician (MD, DO)	11 (73)	–
	Nurse practitioner (NP)	3 (20)	–
	Physician assistant (PA)	1 (7)	–
**Specialty area**			
	Cardiology	–	1 (25)
	Diabetes/endocrinology	–	1 (25)
	Nephrology/renal care	–	1 (25)
	Orthopedics	–	1 (25)

^a^Note: Percentages may total over 100 as PCPs may provide Primary Care services at multiple CBOCs/Medical Centers

### e-Consult Characteristics

Fourteen patients each participated in a single consult, while one patient had two separate e-consults with cardiology and diabetes/endocrinology. Patients had e-consults with diabetes/endocrinology (n=6; 40%), cardiology (n=5; 33%), nephrology/renal care, (n=4; 27%) or neurosurgery (n=1; 7%). On average, PCPs requested 6 (SD 6) e-consults, and VAPHS specialists completed 17 (SD 11) e-consults. PCPs requested e-consults for nephrology/renal care (n=11; 73%), diabetes/endocrinology (n=8; 53%), cardiology (n=7; 47%), neurosurgery (n=6; 40%), and orthopedics (n=3; 20%) (data not shown in tables).

### Satisfaction With the e-Consult Program by Domain

We present descriptive summaries of the *Likert-type satisfaction items* regarding the e-consult process. We also include thematic summaries from our *qualitative* exploration of participant satisfaction with the e-consult process (Table 3). Finally, we present summaries of themes from our exploration of participant perceptions regarding *perceived barriers and facilitators* to the e-consult process. Salient themes will generally reflect the experience of multiple individuals, while views that are expressed by fewer individuals occasionally represent insightful perspectives. We provide examples of participant quotations to further elucidate their responses.

**Table 3:** Codes and frequencies for e-consult satisfaction domains identified during interviews with 15 patients, 15 PCPs, and 4 specialty physicians who utilized the e-consult process.^a^

Codes		# of Patients conveying theme	# of PCPs conveying theme	# of Specialists conveying theme	Total # of participants conveying theme
**Overall satisfaction**					
	1. Communication	8	3	3	14
	2. Timeliness of care	1	7	2	10
	3. Quality of care	4	4	0	8
	4. Travel to VA Pittsburgh Medical Center	1	5	1	7
	5. Experience with e-consults	2	3	1	6
	6. Access to specialist care	0	4	1	5
	7. Electronic medical records system	0	2	1	3
	8. Health-related outcomes	2	0	0	2
	9. Option of face-to-face or e-consult	0	1	0	1
	10. Preferring face-to-face visit	0	0	1	1
	11. Coordination of e-consults	0	1	0	1
	12. e-Consult is easy to use	0	1	0	1
	13. Missing	0	1	0	1
**Quality**					
	1. Quality of care	4	4	0	8
	2. Access to specialist care	0	5	1	6
	3. Timeliness of care	0	5	1	6
	4. Communication	4	1	0	5
	5. Travel to VA Pittsburgh Medical Center	1	2	1	4
	6. Patient satisfaction with care	2	2	0	4
	7. Health-related outcomes	1	1	0	2
	8. Ensure recommendation implementation	0	1	1	2
	9. Experience with e-consults	0	2	0	2
	10. Coordination of e-consults	0	0	1	1
	11. Patient compliance	0	1	0	1
	12. Time required for e-consult	0	0	1	1
	13. Appropriateness of case for e-consult	0	1	0	1
	14. Option of face-to-face or e-consult	0	1	0	1
	15. No answer	3	0	0	3
**Time**					
	1. Time required for e-consult	4	9	4	17
	2. Travel to VA Pittsburgh Medical Center	7	2	0	9
	3. Timeliness of care	2	4	1	7
	4. Communication	1	2	0	3
	5. Electronic medical records system	0	3	0	3
	6. Health-related outcomes	0	1	0	1
	7. Coordination of e-consults	0	0	1	1
	8. No answer	0	1	0	1
	9. Missing	0	1	0	1
**Access**					
	1. Timeliness of care	0	6	2	8
	2. Travel to VA Pittsburgh Medical Center	0	6	2	8
	3. Access to specialist care	4	1	1	6
	4. Communication	5	0	0	5
	5. Clinic time available	1	0	2	3
	6. Appropriateness of case for e-consult	0	1	0	1
	7. Electronic medical records system	1	0	0	1
	8. Add more specialists	0	1	0	1
	9. Option of face-to-face or e-consult	0	1	0	1
	10. No answer	6	2	0	8
**Safety**					
	1. Appropriateness of case for e-consult	0	5	3	8
	2. Quality of care	2	4	1	7
	3. Experience with e-consults	4	2	1	7
	4. Communication	2	3	1	6
	5. Option of face-to-face or e-consult	1	2	1	4
	6. Travel to VA Pittsburgh Medical Center	1	2	0	3
	7. Ensure recommendation implementation	0	0	2	2
	8. Access to specialist care	0	1	0	1
	9. Electronic medical records system	0	1	0	1
	10. No Answer	6	2	0	8
**Expectations**					
	1. No expectations	8	2	1	11
	2. Timeliness of care	3	6	2	11
	3. Quality of care	1	8	0	9
	4. Communication	5	1	1	7
	5. Travel to VA Pittsburgh Medical Center	1	3	0	4
	6. Access to specialist care	2	1	1	4
	7. Improve face-to-face consults	0	0	1	1
	8. e-Consult is easy to use	0	1	0	1
	9. Appropriateness of case for e-consult	0	0	1	1
	10. Somewhat skeptical	1	0	0	1
	11. Experience with e-consults	0	2	0	2
**Confidence**					
	1. Quality of care	7	6	1	14
	2. Experience with e-consults	2	8	0	10
	3. Communication	5	3	1	9
	4. Appropriateness of case for e-consult	1	4	1	6
	5. Timeliness of care	1	2	0	3
	6. Health-related outcomes	1	0	1	2
	7. Travel to VA Pittsburgh Medical Center	0	0	1	1
	8. No answer	0	1	0	1
**Intent to use e-consult in the future**					
	1. Experience with e-consults	0	5	1	6
	2. Quality of care	0	3	2	5
	3. Availability of e-consults	4	0	0	4
	4. Travel to VA Pittsburgh Medical Center	0	3	0	3
	5. Communication	0	1	1	2
	6. Appropriateness of case for e-consult	0	2	0	2
	7. If specialist is asked by PCP	0	0	2	2
	8. Timeliness of care	1	1	0	2
	9. Option of face-to-face or e-consult	0	2	0	2
	10. Electronic medical records system	0	0	1	1
	11. Awareness of e-consults	0	1	0	1
	12. If enough specialist personnel	0	0	1	1
	13. No answer	10	1	0	11

^a^ A given segment of conversation could include one or more codes from each category.

### Overall Satisfaction

Overall, veterans and PCPs were satisfied with the e-consult program, with median (range) Likert ratings of 5.0 (4-5) and 4.0 (3-5) respectively. Specialty physicians reported slightly less overall satisfaction (3.5 [3-5]).

Qualitatively, the most common reason participants from all three groups reported for their overall satisfaction with the e-consults was improved communication (n=14), including effective information transfer, decision making processes, and a patient-centered approach to care (Table 3). Both patients (n=8) and specialists (n=3) identified communication as the domain that was most important regarding their overall satisfaction with the e-consult process. Patient ratings were often related to effective communication with PCPs or providers in general, while specialists largely focused on their effective communication with PCPs. For example, when asked why they were satisfied overall with e-consults, one patient stated, “Well, [my PCP] informed me with answers to my questions,” while a typical specialist quote regarding communication was:

It offers us a chance to talk to the referring physician...and then be certain we have the information that’s required to make the decision.

For PCPs the most common reason for overall satisfaction was timeliness of care (n=7), which included general timeliness of the e-consult process, timeliness of the PCP receiving specialist recommendations, and timeliness of the implementation of specialist recommendations. For example, one PCP stated, “It was prompt, and the patient got the attention they needed in a very reasonable timeline.” Two specialists and only one patient reported timeliness as a reason for overall satisfaction.

### Quality

In general, all participants (veterans, PCPs, and specialty providers) were satisfied with the quality of care provided through the e-consult program (4.0 [3-5], 4.0 [2-5], and 4.0 [3-5] respectively).

The most common reason patients and PCPs reported for their satisfaction with the quality of e-consults was the general quality of care provided (n=8) (Table 3). For example, as one patient stated, “I would get down to the bottom of my problems.” Specialists identified a number of reasons for their rating regarding quality of care, but no domain was mentioned more than once across the specialists.

### Time

Overall, veterans were satisfied with time regarding e-consult (4.0 [3-5]), whereas PCPs and specialty physicians were somewhat less satisfied with time saved with e-consult (4.0 [2-5] and 3.0 [2-5] respectively).

Overall, the discussion was mostly focused on the time required for e-consults (n=17) (Table 3). PCP and specialists’ discussions focused on how e-consults do not save time for health care providers or patients, and sometimes created additional work. For PCPs, the focus was on e-consults creating more work for them:

The preparation…depending on the specialty, what kinds of tests have to be done.

For specialists, discussions focused on:

We didn’t have any set consult time you know at my end to do this…Now there’s an extended figure and it’s intended to go even bigger…I’m planning to increase my hours.

The second most common reason why participants stated that time for e-consults was a reason they were satisfied with e-consults was based on savings on travel to VA Pittsburgh Medical Center (n=9). For example, one veteran stated:

Yeah, I take my pills not only at 7:30 in the morning along with a shot and at 11:30 and then at 3:30 along with a shot there, and then at 11:30, I mean at 10:30. But traveling, it throws you off…and a lot of times you even totally forget it.

### Access

In general, all participants (veterans, PCPs, and specialty providers) were satisfied with improved access to specialty care provided through e-consult (4.0 [3-5], 5.0 [3-5], and 5.0 [4-5] respectively) (data not shown in tables).

The most common reasons for patient satisfaction with access to care were communication (n=5) and access (n=4). The most common reasons for PCP and specialist reported satisfaction regarding access with e-consults was the timeliness of care (n=8, n=2 respectively) and avoiding travel to VA Pittsburgh Medical Center for patients (n=8, n=2 respectively) (Table 3). Regarding timeliness of care, one PCP noted:

Because of the structure and how the e-consult is set up and turn-around time being quicker, it allows us to essentially have an expert opinion in a timely fashion as opposed to a patient being delayed waiting for a traditional consult.

Regarding travel to VA Pittsburgh Medical Center, one specialist noted:

We know that many patients were reluctant to come to Pittsburgh to the specialty clinic because they live two to four hours away…We’re able to provide care to these patients without burdening them with the trip…to Pittsburgh.

Specialists also mentioned that e-consults allows for increased availability of clinic time (n=2). As one specialist stated, “It frees up time for other patients to be seen.”

### Safety

In general, veterans (4.0 [3-5]), PCPs (4.0 [2-5]), and specialty providers (4.0 [3-5]) were satisfied with the safety of the care provided by the e-consult program (data not shown in tables).

Most patients (n=6) reported they were satisfied with the safety of e-consults based on their previous experience with e-consults. The most common reason PCPs (n=5) and specialists (n=3) reported was due to appropriateness of cases for e-consult (Table 3). One specialist noted that “If we have any reservations or the patient has any reservations, we see them [face-to face].”

### Expectations

Overall, veterans (5.0 [3-5]) and PCPs (5.0 [2-5]) were satisfied with their expectations being met with e-consult, whereas specialty physicians were somewhat less satisfied (3.5 [3-5]) (data not shown in tables).

Timeliness of care was the primary expectation mentioned by PCPs (n=6) and specialists (n=2) (Table 3). For example, one specialist stated, “Primary care physicians would know [how] to identify the patients in a timely fashion and then for them to [consult us].”

Most patients did not have any expectations regarding the e-consult (n=8). For example, one patient stated, “I really didn’t know what to expect.”

### Confidence

In general, all participants, including veterans (5.0 [2-5]), PCPs (4.0 [2-5]), and specialty providers (4.5 [3-5]), were confident about care management using e-consult (data not shown in tables).

For patients, the most common reason reported as to why they were confident with e-consults was quality of care (n=7). For example, one patient stated:

Everybody I’ve seen so far, they, they take good care of you and, and if they take a test on me, they’ve given me calls at home to let me know about things.

For PCPs, they largely focused on their previous experience with e-consults (n=8). For example, one PCP stated, “Because I haven’t had any problems so far.” One PCP stated that it was too early in the implementation of the e-consults process to know if he or she was confident about the management of patient care using e-consults.

Specialists did not focus on any one reason in particular; they reported that they were confident with e-consults due to quality of care, communication, appropriateness of case for e-consult, health-related outcomes, and travel to VA Pittsburgh Medical Center.

### Intent to Use e-Consult in the Future

The majority of patients (100%), PCPs (93%), and specialists (100%) indicated that they intended to use e-consults in the future (data not shown in tables).

For patients, the intent to use e-consults in the future focused primarily on quality of care (n=4) and timeliness of care (n=1). As one patient stated regarding e-consults and quality of care, “Well, cause it’s excellent and will lead to better care.”

For PCPs, their intent to use e-consults in the future was largely based on their previous experience with e-consults (n=5). For example, as one PCP stated:

Based on the experiences that I have had so far and the confidence that I have gotten from that, I have no qualms about trying it in the future.

As with patients, specialists also noted that they would use e-consults in the future based on the quality of care (n=2). Specialists also discussed the importance of PCPs referring patients to them (n=2). Therefore, their intention to use e-consults was based on whether PCPs continue to request e-consults.

### Perceptions Regarding Barriers and Facilitators to the e-Consult Process

Below we include brief summaries of themes from our exploration of participant perceptions regarding barriers and facilitators to the e-consult process (Table 4).

**Table 4:** Codes and frequencies for perceived barriers and facilitators to e-consultation utilization identified during interviews with 15 patients, 15 PCPs, and 4 specialty physicians who utilized the e-consult process.^a^

Codes		# of Patients conveying theme	# of PCPs conveying theme	# of Specialists conveying theme	Total # of participants conveying theme
**Barriers**					
	1. Communication	2	6	2	10
	2. Electronic medical records system	0	3	2	5
	3. Time required for e-consults	2	0	1	3
	4. Awareness of e-consults	0	2	1	3
	5. Appropriateness of case for e-consult	0	2	1	3
	6. Specialist credentialing	0	0	2	2
	7. Healthcare provider workload credit	0	0	1	1
	8. Patient confidence in e-consults	0	1	0	1
	9. Coordination of e-consults	0	0	1	1
	10. No answer	11	5	0	16
**Facilitators**					
	1. Communication	8	4	4	16
	2. Quality of care	3	6	0	9
	3. Travel to VA Pittsburgh Medical Center	3	5	0	8
	4. Timeliness of care	2	5	0	7
	5. Electronic medical records system	0	1	4	5
	6. Coordination of e-consults	1	0	2	3
	7. Patient confidence in e-consults	2	0	0	2
	8. No answer	1	3	0	4

^a^ A given segment of conversation could include one or more codes from each category.

### Perceived Barriers

When asked about the things that were not helpful or were barriers to their use of e-consults, most patients did not have an answer (n=11). PCPs (n=8), and specialists (n=2), on the other hand, mentioned communication between PCPs and specialists, as well as between PCPs and patients, as a barrier to e-consult utilization. An equal number of specialists mentioned communication, the electronic medical records system, and specialist credentialing as barriers to the e-consult process (n=2 for each). One PCP stated in reference to patients:

One of our big issues is getting a hold of any of the patients. Their phone numbers have been changed or disconnected or they screen their calls and won’t answer because it comes up unknown name/unknown number.

Other noted perceived barriers for participants included time required for e-consults (n=3), awareness of e-consults (n=3), appropriateness of case for e-consult (n=3), health care provider workload credit (n=1), patient confidence in e-consults (n=1), and coordination of e-consults (n=1).

### Perceived Facilitators

When asked what were some of the facilitators or factors that were helpful regarding the e-consult program, most patients mentioned communication (n=8). As one patient stated, “I got a lot of information for myself, like things that I didn’t really realize that was going on.” Specialists also indicated communication (n=4) and the electronic medical record system (n=4) as facilitators of the use of e-consult. As one specialist stated in regards to the electronic medical record system, “Records are available for [inside] our system, or records from outside of the…VA.” PCPs on the other hand mentioned quality of care (n=6) and travel to VA medical center (ie, saving the patient travel time and money) (n=4) as the main facilitators to the use of e-consult.

Other perceived facilitators included timeliness of care (n=7), the electronic medical records system (n=5), coordination of e-consults (n=3), and patient confidence in e-consults (n=2).

### Addition of Specialty Areas: An Unanticipated Finding

One unanticipated finding was that, without prompting during the interview, 7 PCPs suggested specialty areas to add or that were not appropriate for e-consults. Specialty areas that were suggested included: endocrinology (n=5), rheumatology (n=2), nephrology/renal care (n=2), hematology (n=1), neurology (n=1), urology (n=1), and orthopedics (n=1). It is important to note that, of the aforementioned specialty areas, endocrinology, nephrology/renal care, and neurosurgery were already participating in e-consults. Three of these 7 PCPs also noted areas that they did not think e-consult was appropriate for, including neurosurgery (n=2) and cardiology (n=1). Patients and specialists did not convey such information.

## Discussion

### Principal Findings

This quality improvement evaluation project involved single semistructured telephone interviews conducted with veteran patients, CBOC or rural medical center PCPs, and VAPHS medical center specialty physicians who had used the e-consult process. The project was designed to assess satisfaction with the e-consult process and to identify perceived facilitators of and barriers to e-consult utilization. Our Likert-scale findings showed that, on average, veterans, CBOC and rural medical center PCPs, and VAPHS medical center specialty physicians were satisfied with the e-consult program. Patients were equally satisfied with the areas of quality, time, access, and safety. Both PCPs and VAPHS specialists were most satisfied with access and least satisfied with time. The majority of patients, PCPs, and VAPHS specialists agreed that they intend to use e-consults in the future.

The semistructured interview data revealed that the most common reason patients and specialists reported for their overall satisfaction with e-consults was communication; while timeliness of care was the most common reason for PCPs*.* The most commonly reported domains (as important to satisfaction with e-consults) included timeliness of care and quality of care for patients, timeliness of care and quality of care for PCPs, and communication and the electronic medical records system for specialists. Overall, communication was the most reported perceived barrier and facilitator to use of e-consults.

### Limitations

Our project has several limitations. The project used convenience sampling, a nonprobability sampling technique. The sample was relatively homogeneous in terms of age, gender, race/ethnicity, and treatment site at a veterans’ facility. Due to the timing of the evaluation and the qualitative nature of the project the sample size is small, with participants engaging in a small number of e-consults on which to base their feedback. Therefore, the results may not be representative of or generalizable to all veteran patients, CBOC or rural medical center PCPs, and particularly physician specialists who have completed e-consults. However, the data provide novel information about veteran, CBOC and rural medical center PCP, and VAPHS medical center specialty physician satisfaction with the e-consult process.

### Comparison With Prior Work

While the *Veterans Health Administration* (VHA) has improved access to primary care by establishing CBOCs, access to specialty care continues to be lacking, with upwards of 35%of veterans experiencing issues with access that are directly related to distance and transportation needs [10-12]. Rural veterans have been shown to encounter significant barriers to receiving needed specialty services, particularly routine outpatient specialty services including but not limited to optometry, podiatry, audiology, gynecology, and physical therapy [13]. Over the years, VHA has surveyed the status of veterans and found that distance to VA facilities was one of the reasons veterans cited most frequently for not using VA services such as specialty care [13,14].

It has been argued that technology-based tools and services such as e-consultations with specialty care providers may enable more efficient organization of resources and care provision [10], greater utilization of services [10,15], and improved access to secondary care [16]. According to a recent survey of 440 health care organizations, more than 80% deliver some form of ehealth to patients [13]. Indeed, within the VA, e-consults are just one example of a number of telehealth initiatives implemented across facilities [4]. VHA rapidly adopted this approach nationally, with over half a million e-consults completed in the past few years [17]. On the other hand, e-consult penetration into the private sector and fee-for-service systems has been somewhat limited, perhaps related to billing or payment issues [17].

Similar findings have been reported in other published studies [18-24]; reported levels of patient satisfaction with telemedicine are consistently greater than 80% and frequently at 100% [23], often above the rates of expected satisfaction for traditional forms of health delivery [22]. Primary care providers and specialists have also generally reported quite positive results regarding satisfaction with telemedicine [3,4,18,22]. Overall, patient and provider satisfaction studies indicate optimism for this delivery modality [4,21,23]. Our investigation adds credence to the value of e-consults as it demonstrates that all parties, including veterans, referring PCPs, and VAPHS specialists, are satisfied with e-consults.

### Conclusions

In summary, this quality improvement initiative provides critical veteran and health care provider insights regarding satisfaction with and use of the e-consult program, an innovative approach to specialty care. Our findings suggest that while the reasons for using e-consult may differ, it may be a useful and well-accepted tool to supplement face-to-face specialist visits and to improve health care to patients in rural areas. This information allows us to begin to identify strategies to improve implementation of, and participant satisfaction with the e-consult program.

## Multimedia Appendix 1

Screenshots of CPRS template for a cardiology e-consult.

## Multimedia Appendix 2

Interview guide.

## Abbreviations

CBOC: Community Based Outpatient Clinic
CPRS: Computerized Patient Record System
VA: Veterans Affairs
VAPHS: Veterans Affairs Pittsburgh Healthcare System
VHA: Veterans Health Administration
VISN: Veterans Integrated Service Network
