# Supplementary material for: Veteran, Primary Care Provider, and Specialist Satisfaction With Electronic Consultation
Source: JMIR Med Inform. 2015 Jan 14;3(1):e5. doi: 10.2196/medinform.3725 (PMC4319072; doi:10.2196/medinform.3725)
Supplement: Supplementary file 1 [file medinform_v3i1e5_app1.pdf]

Appendix A. Screenshots of CPRS Template for a Cardiology E-consult

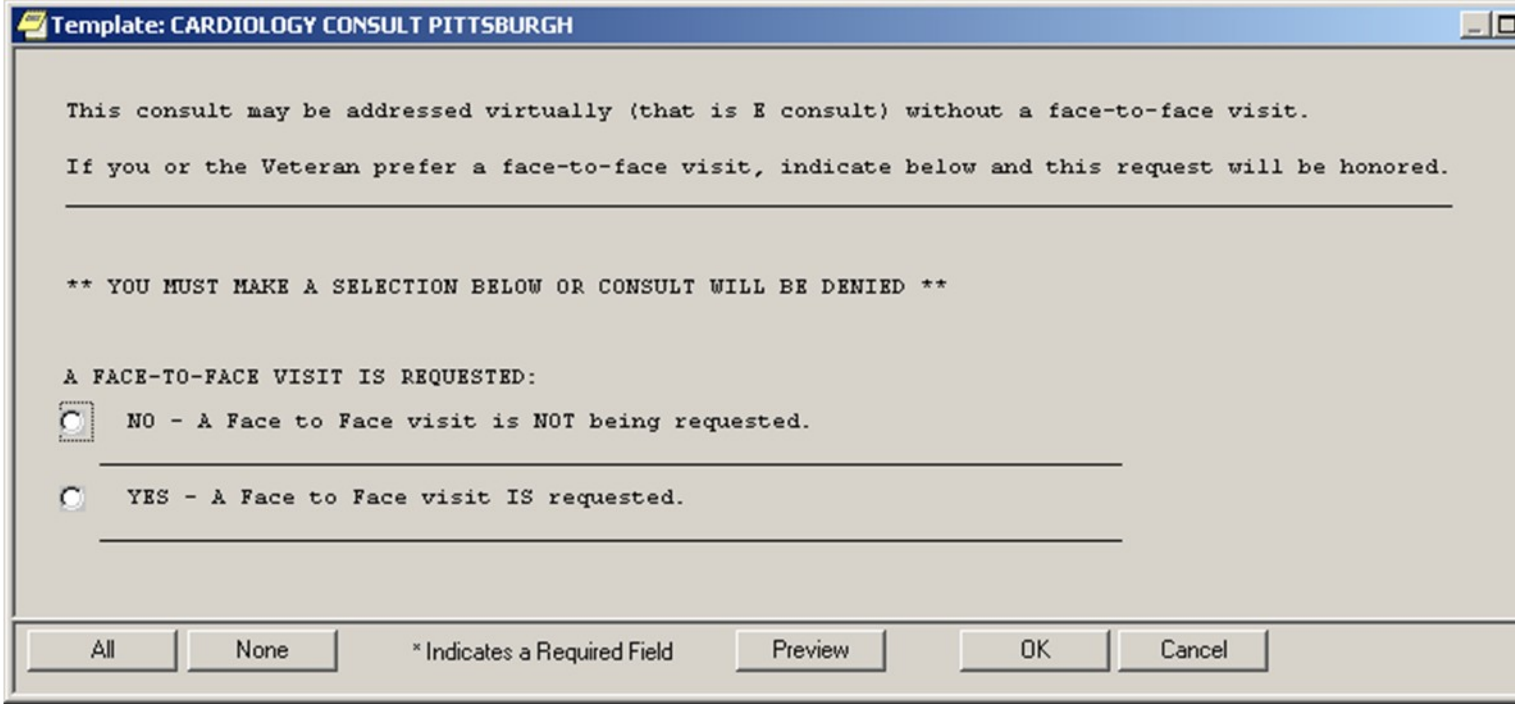

Template: CARDIOLOGY CONSULT PITTSBURGH

This consult may be addressed virtually (that is E consult) without a face-to-face visit.

If you or the Veteran prefer a face-to-face visit, indicate below and this request will be honored.

** YOU MUST MAKE A SELECTION BELOW OR CONSULT WILL BE DENIED **

A FACE-TO-FACE VISIT IS REQUESTED:

(•) NO - A Face to Face visit is NOT being requested.

( ) SELECT THIS BUTTON TO CONTINUE
****
CATEGORY: IN PROCESS STATUS: PENDING; ELIGIBILITY STATUS IS UNVERIFIED PRIORITY:

Is the reason for this consult because of a Service Connected condition?
*( ) Yes. ( ) No. ( ) Not sure.

****
(ORDERING PROVIDER CREDENTIALS ARE REQUIRED)
Ordering Provider: *
Credentials: (Select from the list in the box or type in the box) *
Contact Number(s): *
Best time to contact Provider: *

==
Medications dispensed from VA facilities.
Non VA medications as documented and/or reported by patient/surrogate:
==
Active Inpatient and Outpatient Medications (including Supplies):

No Medications Found

==
No Active Remote Medications for this patient
==

ZZMOUSE,X
UNKNOWN
Patient Phone: Phone number Unknown
(Other phone numbers - Cell, work, etc.)

*

All | None | * Indicates a Required Field | Preview | OK | Cancel

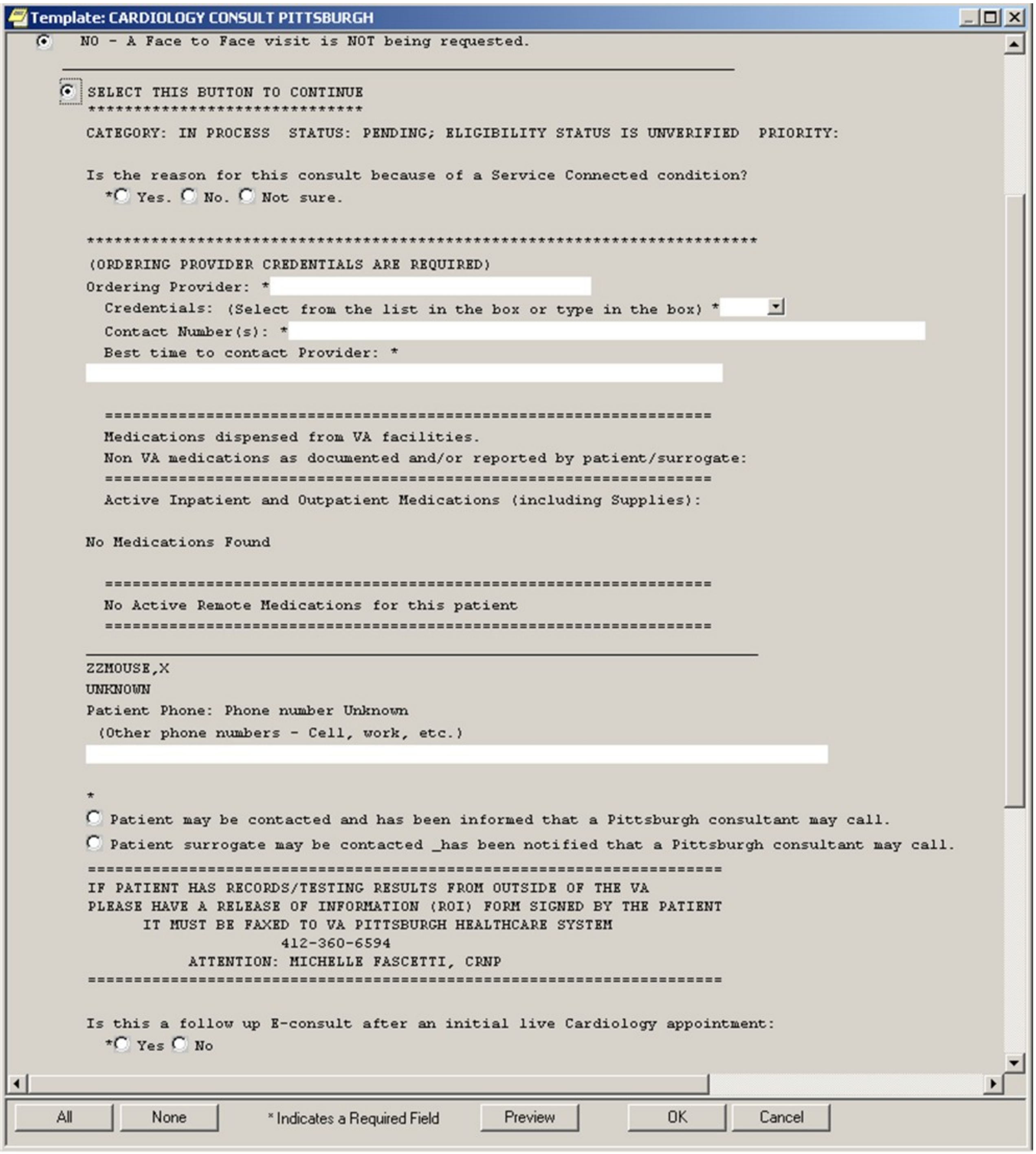
Template: CARDIOLOGY CONSULT PITTSBURGH

NO - A Face to Face visit is NOT being requested.

SELECT THIS BUTTON TO CONTINUE
****
CATEGORY: IN PROCESS STATUS: PENDING; ELIGIBILITY STATUS IS UNVERIFIED PRIORITY:

Is the reason for this consult because of a Service Connected condition?
*Yes. No. Not sure.

****
(ORDERING PROVIDER CREDENTIALS ARE REQUIRED)
Ordering Provider: *
Credentials: (Select from the list in the box or type in the box) *
Contact Number(s): *
Best time to contact Provider: *

==
Medications dispensed from VA facilities.
Non VA medications as documented and/or reported by patient/surrogate:
==
Active Inpatient and Outpatient Medications (including Supplies):

No Medications Found

==
No Active Remote Medications for this patient
==

ZZMOUSE,X
UNKNOWN
Patient Phone: Phone number Unknown
(Other phone numbers - Cell, work, etc.)

*
Patient may be contacted and has been informed that a Pittsburgh consultant may call.
Patient surrogate may be contacted _has been notified that a Pittsburgh consultant may call.
==
IF PATIENT HAS RECORDS/TESTING RESULTS FROM OUTSIDE OF THE VA
PLEASE HAVE A RELEASE OF INFORMATION (ROI) FORM SIGNED BY THE PATIENT
IT MUST BE FAXED TO VA PITTSBURGH HEALTHCARE SYSTEM
412-360-6594
ATTENTION: MICHELLE FASCETTI, CRNP
==

Is this a follow up E-consult after an initial live Cardiology appointment:
*Yes No

All | None | * Indicates a Required Field | Preview | OK | Cancel

Template: CARDIOLOGY CONSULT PITTSBURGH

Non VA medications as documented and/or reported by patient/surrogate:

===

Active Inpatient and Outpatient Medications (including Supplies):

No Medications Found

===

No Active Remote Medications for this patient

===

ZZMOUSE,X

UNKNOWN

Patient Phone: Phone number Unknown

(Other phone numbers - Cell, work, etc.)

*

- ( ) Patient may be contacted and has been informed that a Pittsburgh consultant may call.
- ( ) Patient surrogate may be contacted _has been notified that a Pittsburgh consultant may call.

==

IF PATIENT HAS RECORDS/TESTING RESULTS FROM OUTSIDE OF THE VA
PLEASE HAVE A RELEASE OF INFORMATION (ROI) FORM SIGNED BY THE PATIENT
IT MUST BE FAXED TO VA PITTSBURGH HEALTHCARE SYSTEM
412-360-6594
ATTENTION: MICHELLE FASCETTI, CRNP

==

Is this a follow up E-consult after an initial live Cardiology appointment:

*( ) Yes ( ) No

There are *

- ( ) NO relevant records, procedures or testing results done outside of the VA.
- ( ) All relevant testing results available in VA records.
- ( ) procedure/test results from outside the VA. Signed ROI form will be faxed to 412-360-6594 (Att:

REASON FOR REQUEST INCLUDING PERTINENT FINDINGS & MEDICAL HISTORY:

*

(SELECT LINK BELOW TO OBTAIN RELEASE OF INFORMATION CONSENT)

https://vaww.visn4.portal.va.gov/pittsburgh/home/has/HIMS/Shared%20Documents/Release%20of%20Info%20I

**** END ****

END OF TEMPLATE YOU DO NOT HAVE TO GO ANY FURTHER

( ) YES - A Face to Face visit IS requested.

All | None | * Indicates a Required Field | Preview | OK | Cancel
