# Supplementary material for: Veteran, Primary Care Provider, and Specialist Satisfaction With Electronic Consultation
Source: JMIR Med Inform. 2015 Jan 14;3(1):e5. doi: 10.2196/medinform.3725 (PMC4319072; doi:10.2196/medinform.3725)
Supplement: Supplementary file 2 [file medinform_v3i1e5_app2.pdf]

Appendix B: Interview Guide.

INTRODUCTION

I will start out by asking you some questions about yourself. Then I will ask you about your experience with the VA electronic consultation program, or e-consults. An e-consult is an opportunity for a Veteran and his or her primary care provider from a Community Based Outpatient Clinic to request a consultation from the VA Pittsburgh Healthcare System for specialty care, without requiring a face-to-face encounter between a Veteran and specialist.

We're very interested in knowing about both good qualities and problems or difficulties that you may have experienced with e-consult. We will focus first on good qualities, or "facilitators," and problems, or "barriers," to using the VA e-consult. Then, I will ask you some questions about your satisfaction with the e-consult process.

It is important for you to know that there are no "right or wrong" answers, and all of the information you share with us will be kept private, in that your name or other identifying information will not be linked to any of your specific responses. Before we begin, do I have your verbal permission to audio-record our conversation?

Do you have any questions before we begin the interview? Okay, great.

DEMOGRAPHICS

I'm going to start by asking you some questions about yourself.
[Ask questions from Demographic Questionnaire].

I'm going to turn on the audio-recorder now, OK?
[Turn on audio-recorder]

USE OF E-CONSULT

Now, I'd like to ask you about your experience with the VA e-consult.

- Please tell me in your own words about your experience with VA e-consults.

FACILITATORS

- What are some of the factors or things were helpful, or facilitated your use of the e-consult?

- Given all of the things we have just talked about, which facilitator is most significant to your use the e-consult?

BARRIERS

- What are some of the things that were not helpful or were barriers to your use of the e-consult?

- Given all of the things we have just talked about, which barrier is the biggest problem to use of the e-consult?

- Given all of the things that we have just talked about, what are the major things the VA could do that would improve using e-consults?

SATISFACTION

Now, I'd like to talk about your satisfaction with e-consults. I'm going to ask you whether you agree or disagree with some statements.

Overall Satisfaction

- The first statement is: I am satisfied with e-consults. Would you say you (insert options).
  _____Completely Disagree
  _____Disagree
  _____Neither Agree Nor Disagree
  _____Agree
  _____Completely Agree

- When you think overall about e-consults, what are some of the good qualities that come to mind?

- On the other hand, when you think overall about e-consults, what are some of the poor qualities that come to your mind?

Quality

- The next statement is: Using e-consults results in quality patient care. When I say quality, I mean the degree to which the health services [you] received increase the likelihood of [your] desired health outcomes and are consistent with current professional knowledge. Would you say you (insert options).
  _____Completely Disagree
  _____Disagree
  _____Neither Agree Nor Disagree
  _____Agree
  _____Completely Agree

- What would you say are some important things that make you respond this way about the quality of patient care with e-consults?

Time

- The next statement is: Using e-consults saves me time. Would you say you (insert options).
  _____Completely Disagree
  _____Disagree

_____Neither Agree Nor Disagree
_____Agree
_____Completely Agree

- What would you say are some important things that make you respond this way about your time with e-consults?

Access

- The next statement is: E-consults improve(s) [patient/my] access to specialty care. Would you say you (insert options).
  _____Completely Disagree
  _____Disagree
  _____Neither Agree Nor Disagree
  _____Agree
  _____Completely Agree

- What would you say are some important things that make you respond this way about patient's access to specialty care with e-consults?

Safety

- The next statement is: Managing [a patient's/my] care through an e-consult can be done safely. Would you say you (insert options).
  _____Completely Disagree
  _____Disagree
  _____Neither Agree Nor Disagree
  _____Agree
  _____Completely Agree

- What would you say are some important things that make you respond this way about safety with e-consults?

Expectations

- The next statement is: My expectations were met by using e-consults. Would you say you (insert options).
  _____Completely Disagree
  _____Disagree
  _____Neither Agree Nor Disagree
  _____Agree
  _____Completely Agree

- What would you say are some important things that make you respond this way about your expectations with e-consults?

Confidence

- The next statement is: I am confident about the management of [my] care using e-consult. Would you say you (insert options).

_____Completely Disagree
_____Disagree
_____Neither Agree Nor Disagree
_____Agree
_____Completely Agree

- What would you say are some important things that make you respond this way about the management of care with e-consults?

Intention to Use

- The next statement is: I intend to use e-consults in the future [when offered as an option by my PCP]. Would you say you (insert options).
  _____Completely Disagree
  _____Disagree
  _____Neither Agree Nor Disagree
  _____Agree
  _____Completely Agree

- What would you say are some important things that make you respond this way about your intent to use e-consults in the future?

Satisfaction Summary

- Given all of the things we have just talked about, which one thing is the most important to your satisfaction with e-consults?

- Given all of the things that we have just talked about, what are the major things the VA could do that would improve your satisfaction with e-consults?

- Are there any other things about your satisfaction with e-consults that you think we should know about that we have not discussed?

CLOSING

In closing, is there anything else you would like to add that we did not discuss?
[Turn off audio-recorder]

Thank you so much for your time in discussing these matters with us. Do you have any questions?

Thanks again for taking your time to speak with us. Have a nice day.
